# An Assessment of the Prevalence and Risk Factors of Hypertensive Crisis in Patients Who Visited the Emergency Outpatient Department (EOPD) at Adama Hospital Medical College, Adama, Oromia, Ethiopia: A 6-Month Prospective Study

**DOI:** 10.1155/2024/6893267

**Published:** 2024-04-29

**Authors:** Abel Tezera Abebe, Yabets Tesfaye Kebede, Bekri Delil Mohammed

**Affiliations:** School of Medicine, Faculty of Medical Sciences, Institute of Health, Jimma University, Jimma, Ethiopia

## Abstract

**Background:**

Over 1 billion people worldwide suffer from the common chronic medical condition of hypertension. A hypertensive crisis occurs when blood pressure exceeds 180/110 mmHg. Depending on whether the target organ is harmed, the situation may be presented as a hypertensive emergency or urgency.

**Objective:**

To assess the prevalence and risk factors of patients with hypertensive crises who visited the Emergency Outpatient Department (EOPD) at Adama Hospital Medical College in Adama, Oromia, Ethiopia, between January 01 and August 31, 2021, G.C. *Methodology*. A cross-sectional, prospective study on the hypertensive crisis was conducted at Adama Hospital Medical College from January 01 to August 31, 2021, G.C. The data were collected using a standardized questionnaire, validated for completeness, and analyzed using SPSS. The findings were tabulated, and conclusions and recommendations were conveyed.

**Result:**

Out of 9,082 patients who visited the EOPD during the six-month period, 444 individuals with hypertensive crises were identified, representing a prevalence of 4.9%. Of these, 56.8% were men, resulting in a *M* : *F* ratio of 1.31 : 1. Those between the ages of 66 and 75 were the most affected. At presentation, 91.0% of the study participants were known hypertensive patients. Among the known hypertensive patients, the majority (34.9%) were known to have been hypertensive for 5–10 years. Of the known hypertensive patients, 48.6% were found to be adherent. Hypertensive urgency was discovered to be far more common than hypertensive emergencies (63.5% vs. 36.5%). Headache was the most common presenting symptom, and most patients (36.5%) presented to the health setup in less than 24 hours. The main risk variables identified were drug discontinuation, family history of hypertension, salt consumption, and alcohol usage. The main excuse for the lack of adherence was the cost of the medications. More than half of the patients do not have any additional comorbidities, and of those who do, diabetes mellitus is the most prevalent. A stroke was identified as a major complication. *Conclusions and Recommendations*. Hypertensive crises are one of the most prevalent reasons for EOPD admission and are linked to significant consequences. At presentation, most of the study subjects were known hypertension patients. Diabetes mellitus was discovered to be a comorbid condition in one-quarter of them. Although more than half of the patients had improved, the death rate still remained high. Infrastructure and capacity building to provide hospitals with the requisite baseline investigations are among the government's recommendations. Health practitioners are expected to make improvements, such as by educating the public about the need for lifestyle changes and evaluating and managing any hypertension problems.

## 1. Introduction

### 1.1. Background Information

One of the primary causes of the global disease burden is hypertension. Over a billion people worldwide have high blood pressure, which is considered the cause of 9.4 million annual deaths. Cardiovascular diseases such as coronary heart disease, congestive heart failure, ischemic and hemorrhagic strokes, renal failure, and peripheral arterial disease are all twice as likely to occur in people with hypertension. Although antihypertensive medications lower the risk of cardiovascular and renal disease, a sizable portion of hypertension sufferers go untreated or receive insufficient care.

Given that hypertension is a major global public health concern, its prevention, detection, treatment, and control should be given top priority. However, the majority of countries in Sub-Saharan Africa (SSA) are still battling infectious diseases such as HIV, malaria, and tuberculosis, and the majority of their governments lack adequate funding for healthcare [1]. A rise in the prevalence of hypertension in this area is therefore likely to have negative effects.

Previously, the Joint National Committee classified hypertension into four stages: normal (less than 120/80 mmHg), pre-HTN (between 120 and 139/80 and 89 mmHg), stage I (between 140 and 159/90 and 99 mmHg), and stage II (above 160/100 mmHg). The new ACC/AHA guidelines, the first comprehensive set since 2003, changed the definition of high blood pressure to account for complications that can arise at lower readings and to enable earlier intervention. BP categories according to the new ACC/AHA guideline are normal (below 120/80 mmHg), elevated (SBP between 120 and 129 and DBP below 80 mmHg), stage 1 (SBP between 130 and 139 and DBP between 80 and 89 mmHg), and stage 2 (SBP at least 140 or DBP at least 90 mmHg).

The Eighth Joint National Committee [2] categorizes blood pressure elevations as either “hypertensive urgencies,” which include asymptomatic severe hypertension without target organ damage, or “hypertensive emergencies,” which include acute, life-threatening conditions associated with marked increases in blood pressure, typically 180/120 mmHg. It is crucial to distinguish between hypertensive emergencies and urgency when developing a treatment plan. The former seeks to lower blood pressure gradually over several hours to days to 160/100 mmHg, whereas the latter seeks to lower blood pressure right away (although not necessarily to normal ranges) to prevent or limit the deterioration of the target organ [3, 4].

It should be noted that acute end-organ damage, rather than a specific blood pressure level, distinguishes hypertensive urgency from an emergency. A hypertensive emergency is a clinical diagnosis, and the clinical condition of the patient is more significant than the blood pressure's absolute value [3].

The phrase “hypertensive emergency” has replaced the term “malignant hypertension” in national and international blood pressure control recommendations. Malignant hypertension was once used to describe a condition characterized by elevated blood pressure combined with encephalopathy or acute nephropathy [2].

### 1.2. Statement of the Problem

The high incidence of hypertension and the risk of cardiovascular disease it carries make it a major public health concern worldwide [5]. The global prevalence of hypertension is expected to exceed 29% by 2025. In Sub-Saharan Africa (SSA), approximately 75 million people (roughly twice the population of California) have hypertension, with a projected 125.5 million people affected by 2025 [6], and more than 80% of deaths from hypertension and associated cardiovascular diseases occur in low- and middle-income countries [7].

In the industrialized world, hypertension is becoming a more common health condition due to an extended lifespan and the prevalence of risk factors such as obesity, inactivity, and poor diet. The frequency is already comparable to that seen in wealthy nations and many emerging nations, especially in urban cultures [8].

Uncontrolled hypertension continues to be a major contributor to cardiovascular disease, despite the availability of effective treatments [9]. As a result, when systolic blood pressure to diastolic blood pressure (SBP/DBP) is greater than 180/120 mmHg, suboptimal BP control might result in a hypertensive crisis [10].

The treatment of hypertension has been linked to a 40% reduction in the risk of stroke and a 15% reduction in the risk of myocardial infarction. Even though HTN medication has been demonstrated to prevent CVD and to extend and improve life, hypertension is still poorly treated around the world [8]. Furthermore, obesity, diabetes, hyperlipidemia, and tobacco use are all common cooccurring cardiovascular risk factors with hypertension, all of which raise the cardiovascular risk related to hypertension. Insufficient attention is given to these coexisting risk factors in hypertensive patients around the world, which has a high morbidity rate and mortality rate.

Over the past 40 years, hypertensive crises have become more common, despite the development of increasingly effective antihypertensive medications [11]. Given that most hypertensive crises involve people with chronic hypertension, this may be related to a lack of awareness on the part of the society. This is mainly because people do not take their medications as prescribed; they get high on stimulants such as cocaine; and they experience withdrawal symptoms from antihypertensive medications such as clonidine and beta blockers.

Although it is well known that treating hypertensive crisis quickly is necessary to avoid permanent or worsened target organ damage [9], this does not seem to apply in our setting because of a number of factors, including gaps in patients' full clinical history, inadequate laboratory and radiographic equipment, and a lack of blood pressure cuffs that are of the right size, which is crucial because using a cuff that is too small for the arm size has been shown to artificially elevate BP.

There is a lack of data on the types of hypertensive crises seen in Sub-Saharan Africa, as well as the symptoms and outcomes. Most notably in Ethiopia, little is known about the prevalence, risk factors, and prognosis of the hypertensive crisis in East Africa. Data on these tendencies are critical for increasing physician awareness. Thus, raising awareness of the community's risk factors will aid in lowering morbidity and mortality rates among these hypertensive patients.

### 1.3. Significance of the Study

According to numerous studies, the majority of people with hypertensive crises who stopped taking their medication for a significant period of time did so because they felt better. This result demonstrates the significance of effective hypertension control in reducing high blood pressure-related complications.

Health professionals in our situation would be better able to assess the severity of the issue and develop effective solutions if they were aware of the risk factors for hypertensive crisis. It will serve as a starting point for additional research in the field by a number of governmental and nongovernmental organizations involved in the management of noncommunicable diseases such as hypertension. The study will offer a helpful manual for people to follow in order to lessen the financial, social, psychological, and physical effects of hypertension.

This research could therefore aid in improving the management and rehabilitation of hypertensive patients as well as the prevention of the emergence of chronic hypertension complications, primarily brought on by patients' poor adherence to hypertensive regimens and insufficient care and follow-up for the condition.

## 2. Objectives

### 2.1. General Objectives

- To assess the prevalence and risk factors of hypertensive crisis among patients who visited the Emergency Outpatient Department (EOPD) of Adama Hospital Medical College, 2021 G.C.

### 2.2. Specific Objectives

- To assess the sociodemographic characteristics of patients with hypertensive crises.
- To assess the risk factors for hypertensive crisis among patients who visited the EOPD at Adama Hospital Medical College (AHMC), 2021, G.C.
- To assess the complications of hypertensive crisis among patients who visited the EOPD at AHMC, 2021, G.C.

## 3. Methodology

### 3.1. Study Area

Adama Hospital Medical College (AHMC) was previously known by the names Haile Mariam Mamo Memorial Hospital and Adama Referral Public Hospital at different times. It is one of the first medical hospitals situated in Adama town, located in the Oromia region, 100 kilometers (about 62.14 miles) southeast of Addis Ababa, Ethiopia. The hospital was inaugurated by missionaries from abroad in 1938 E.C. and was among the first nongovernmental hospitals in the nation.

The hospital was upgraded to a medical college in 2003 EC because of its location, patient load, and staff capacity. The hospital serves a catchment population of more than 6 million from five regions (Oromia, Amhara, Afar, Somalia, and Dire Dawa). The hospital has 232 beds and serves 1,000 patients daily through six medical case teams (OPD) and various specialty clinics.

### 3.2. Study Period

The study period was from January 01 to August 31, 2021, G.C.

### 3.3. Study Design

A prospective institution-based cross-sectional study was conducted.

### 3.4. Source Population

The source populations of the study were patients who presented with hypertensive crisis to the EOPD at Adama Hospital Medical College (AHMC) from January 01 to August 31, 2021, G.C.

### 3.5. Study Population

The study population consisted of any selected patient with hypertension who met the study's inclusion criteria.

### 3.6. Inclusion Criteria

All adult patients over the age of 18 who presented with hypertensive crises to the EOPD at Adama Hospital Medical College during the study period met the inclusion criteria.

### 3.7. Exclusion Criteria

Any patient who was younger than 18 years old and had incomplete data or those who self-discharged themselves after being seen at the EOPD were excluded from the study.

### 3.8. Sample Size and Sampling Technique

Sampling was not used because all cases of patients diagnosed with hypertensive crises after presenting to the hospital during the previously mentioned study period were included.

### 3.9. Dependent Variables

- Prevalence of hypertensive crisis.

### 3.10. Independent Variables

- Sociodemographic variables such as age, sex, educational status, and income.
- History of hypertension.
- Family history of hypertension.
- Alcohol abuse and cigarette smoking.
- Salt consumption.
- Other comorbid illnesses such as diabetes, cardiac failure, or renal failure.

### 3.11. Data Collection Procedure

A closed-ended, structured questionnaire was employed for data collection. This tool was meticulously developed through a review of the relevant literature [12–15] and subsequently pretested on 5% of the sample size, consisting of 22 patients. Following this pretest, necessary amendments were made to the data abstraction tool to ensure its effectiveness.

The data collection process involved gathering information on various aspects, including patient details, clinical staging of the disease at diagnosis, treatment duration, drug specifics, and the results of investigations conducted.

The task of data collection was carried out by the principal investigator and four medical interns assigned to the department. Before commencing data collection, all involved personnel underwent comprehensive training, ensuring a consistent approach to data gathering. Throughout the data collection period, supervision was provided by the principal investigator daily to maintain the quality and accuracy of the collected data.

### 3.12. Data Quality Control

To ensure the completeness, accuracy, and consistency of data collection, one hour of training was given to the data collectors.

Data editing was conducted daily by the principal investigator to check for accuracy, consistency, and completeness. The data were entered and cleaned by the principal investigator before analysis.

### 3.13. Data Processing and Analysis

After collecting, cleaning, and checking the data for completeness by the principal investigator, it was analyzed using SPSS version 26 software, and the results were expressed using appropriate frequency distribution and cross-tabulation for the selected variables.

Associations between the independent and dependent variables were tested using the odds ratio, and a 95 percent confidence interval was used to measure the strength of the association between the independent and dependent variables.

### 3.14. Operational Definitions

*Prevalence* represents the total number of cases.
*Incidence represents* the number of new cases. It is a better indicator of the evolution of the epidemic, as it accounts only for the new cases, while prevalence also accounts for deaths related to the case.
*Hypertension* is defined by the average of two systolic blood pressures (SBPs) between 130 and 139 and a diastolic blood pressure (DBP) between 80 and 89 mmHg and/or the current use of antihypertensive medications at the time of admission [2].
*Severe hypertension* is defined as an average systolic blood pressure (SBP) of ≥180 and/or a diastolic blood pressure (DBP) of ≥120 mmHg.
*Hypertensive emergency* is severe hypertension associated with end-organ damage [2].
 

*Acute kidney injury* is currently defined by a rise in serum creatinine concentration from the baseline of at least 0.3 mg/dL within 48 hours or at least 50% higher than baseline within 1 week, or a reduction in urine output to below 0.5 mL/kg per hour for longer than 6 hours [8].
*Acute myocardial infarction* was defined according to the previous World Health Organization's criteria for acute, evolving, or recent myocardial infarction, which requires a combination of two of three characteristics: typical symptoms (i.e., chest discomfort), a typical rise and a gradual fall of troponin or a more rapid rise and fall of CK-MB, and ECG changes indicative of ischemia (ST segment elevation or depression) involving the development of pathological Q-waves [16].
*Hypertensive encephalopathy* is a life-threatening condition marked by a high blood pressure, resulting in symptoms such as changes in mental state, severe headaches, dizziness, vision disturbances, and seizures [17].
 

*Acute pulmonary edema* is defined as an increase in the extravascular fluid content of the lungs resulting in dyspnea and bilateral basal crackles confirmed by a chest X-ray [18, 19].
*Hypertensive retinopathy* describes a spectrum of microvascular abnormalities in individuals with high blood pressure and can be categorized into different stages [20] as follows:
    Mild: retinal arteriolar narrowing, wall thickening or opacification, and arteriovenous nicking (nipping).
    Moderate: hemorrhages, either flame or dot-shaped, cotton-wool spots, hard exudates, and microaneurysms.
    Severe: some or all of the above, as well as papilledema.
*Hypertensive stroke*, also known as hemorrhagic stroke, is due to a blood vessel rupture in the brain, resulting in the sudden onset of neurological deficits [21].

### 3.15. Ethical Consideration

A proposal for the study format was submitted to the Jimma University Department of Medicine Office to get work approval for the study. The Ethics Committee of Jimma University gave ethical approval for the study. After approval, an official letter was obtained from the Department of Medicine at Jimma University to get permission from Adama Hospital Medical College.

Each respondent was informed about the objective, purpose, and assurance of confidentiality. Before starting data collection, informed verbal consent was obtained from each subject. Patient identification, healthcare provider information, and drug product information were kept private, as their names were not displayed in the questionnaire or final report (available here).

## 4. Results

### 4.1. Sociodemographic Characteristics

During the study period, a total of 444 cases of hypertensive crisis were seen out of 9082 patients who visited the EOPD (4.9%), of which, 56.8% were men and the rest, 43.2%, were women. The most affected age groups were 66–75 years (42.8%) and 56–65 years (23.4%), followed by the age group of >75 years, which accounted for 17.6% of the cases. 78.2% of the study subjects were from urban areas, and the rest, 21.8%, were from rural areas (Table 1).

**Figure 1:**
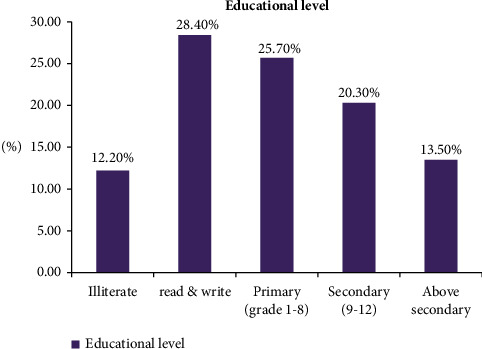
Educational level of hypertensive crisis patients presented to the EOPD at AHMC, Adama, Oromia, Ethiopia, from January 01 to August 31, 2021, G.C.

**Figure 2:**
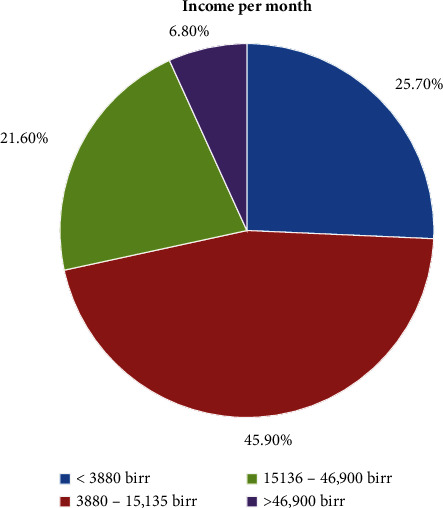
Income per month of patients with hypertensive crisis presented to the EOPD at AHMC, Adama, Oromia, Ethiopia, from January 01 to August 31, 2021, G.C.

Of the study subjects, 28.4% were able to read and write, followed by 25.7% who had attended primary school (Figure 1). Around 45.9% of the patients had an average income per month of 3,880–15,135 birr, followed by 25.7% who earned 3,880 birr per month (Figure 2).

### 4.2. Clinical Status of Patients with Hypertensive Crises at Presentation

At presentation, 91.0% of the study participants were known hypertensive patients, while 9.0% had just received a diagnosis. Of the known hypertensive patients, the majority (33.8%) were known to have been hypertensive for 5–10 years. Looking at the status of drug adherence, 48.6% were found to be adherent, and 234 (52.7%) were on follow-up (Table 2).

Among patients who were not on the follow-up, the primary reason for their absence from the follow-up appointments was a lack of knowledge, accounting for 70 cases (40.3%). This was followed by residing too far from the health center and negligence, each cited in 34 cases (19.5%). In addition, concerning patients who did not adhere to their prescribed medications, the predominant reasons for nonadherence were the high cost of medications and a perceived sense of well-being without taking the medication, each identified in 28.1% of the cases (Table 3).

Most of the study subjects complained of headaches (43.9%), body weakness (27.7%), incidental findings on follow-up (12.0%), and loss of consciousness (8.1%). The duration of the complaint in most of the cases was less than a day (36.5%), followed by 24–72 hours (27.2%) (Table 4).

### 4.3. Factors Associated with Hypertensive Crisis

**Figure 3:**
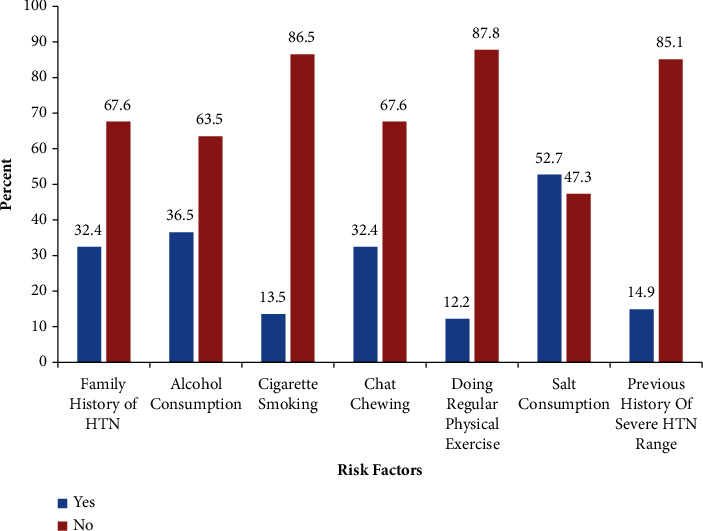
Risk factors of patients with hypertensive crisis presented to the EOPD at AHMC, Adama, Oromia, Ethiopia, from January 01 to August 31, 2021, G.C.

Risk factors linked with hypertensive crisis among patients at the EOPD comprise salt consumption in 52.7% of the cases, alcohol consumption in 36.5% of the cases, a family history of hypertension in 32.4% of the cases, chewing chat in 32.4% of the cases, and a prior history of severe hypertension in 14.9% of the cases (Figure 3).

**Figure 4:**
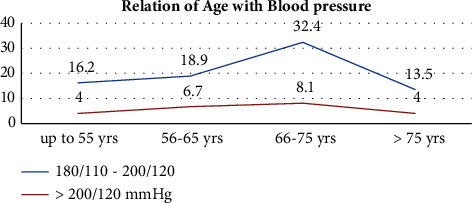
Relationship of age and blood pressure among patients with hypertensive crisis presented to the EOPD at AHMC, Adama, Oromia, Ethiopia, from January 01 to August 31, 2021, G.C.

Age was significantly associated with severe hypertension in the univariate analysis using the Pearson's chi-square test of sociodemographic variables. The majority of patients with BP > 200/100 mmHg were >56 years old, with the proportion increasing as age advances compared to those between 45 and 55 (*P* value = 0.004) (Figure 4).

**Figure 5:**
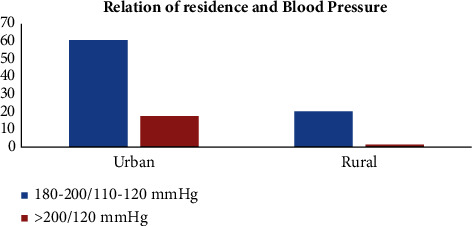
Relationship of residence and hypertension among patients with hypertensive crisis presented to the EOPD at AHMC, Adama, Oromia, Ethiopia, from January 01 to August 31, 2021, G.C.

Similarly, when compared with those from rural areas, those from urban areas are more likely to present with severe hypertension than those from rural areas (*P*=0.01) (Figure 5).

**Figure 6:**
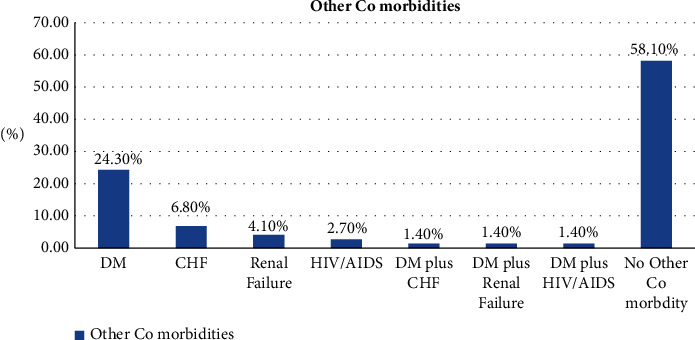
Other comorbidities of patients with hypertensive crisis presented to the EOPD at AHMC, Adama, Oromia, Ethiopia, from January 01 to August 31, 2021, G.C.

Associated comorbid illness was found among patients with hypertensive crises presenting to the emergency department, including diabetes mellitus in 108 (24.3%) of the cases, CHF in 30 (6.8%), renal disease in 24 (4.1%), and 6 (1.4%) of them had DM and CHF, and another 6 (1.4%) had DM and renal failure simultaneously, but there were no other identified comorbid illnesses in the majority of the cases (258, or 58.1%) (Figure 6).

### 4.4. Physical, Laboratory, and Radiological Findings among Patients with Hypertensive Crisis

During physical examination, patients presenting with hypertensive crises initially showed SBP ranging from 180 to 190 mmHg and DBP ranging from 110 to 115 mmHg in 222 cases (50.0%). This was followed by SBP ranging from 191 to 200 mmHg and DBP ranging from 116 to 120 mmHg in 137 cases (30.9%), while 24 cases (5.4%) had SBP greater than 220 mmHg and DBP above 130 mmHg. In terms of BMI, 199 (44.8%) of the study population had a BMI between 18.5 and 24.9 kg/m^2^, with 107 (24.1%) having a BMI between 25 and 29.9 kg/m^2^ (Table 5).

**Figure 7:**
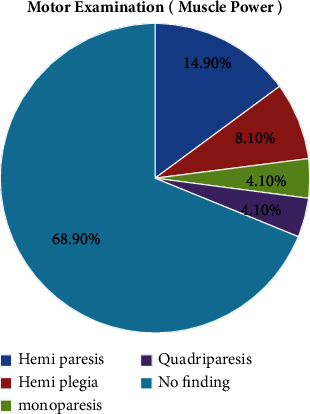
Motor examination (muscle power) of patients with hypertensive crisis presented to the EOPD at AHMC, Adama, Oromia, Ethiopia, from January 01 to August 31, 2021, G.C.

Regarding the neurologic examination, the majority of patients, i.e., 270 (60.8%) had a GCS of 15/15, while 54 (18.9%) had a GCS of 8–12 and 36 (8.1%) had a GCS of less than 8. On motor examination, 66 (14.9%) had hemiparesis, followed by 36 (8.1%) with hemiplegia. In 306 (68.9%) of the patients, there were no findings (Figure 7).

On laboratory evaluation, the random blood sugar values were as follows: in more than half of the patients (74.5%), the random blood sugar values were in the range of 70–110 mg/dl; in 13.3% of the patients, the RBS was 111–200 mg/dl; and in 5.4% of the patients, the RBS was >200 mg/dl. On serologic examination, serum creatinine values were in the range of 0.7–1.2 mg/dl in 366 (82.4%) cases, 1.2–2 mg/dl in 53 (11.9%) cases, and in 25 (5.6%) of the cases, serum creatinine was above 2 mg/dl (Table 6)

Most of them had normal chest X-ray findings on radiographic examination: 252 (51.8%) had normal chest X-ray findings and 60 (13.6%) had abnormal chest X-ray findings. ECGs were performed on 414 (93.2%) of the patients, and the ECGs were normal in 378 (85.1%), with only 12 (2.7%) of the patients showing STEMI. A CT scan of the head was performed on 204 (45.99%) of the patients, the majority of whom had a hemorrhagic stroke (12.6%). A CT scan of the head was not performed on 54.1% of the patients (Table 7).

**Figure 8:**
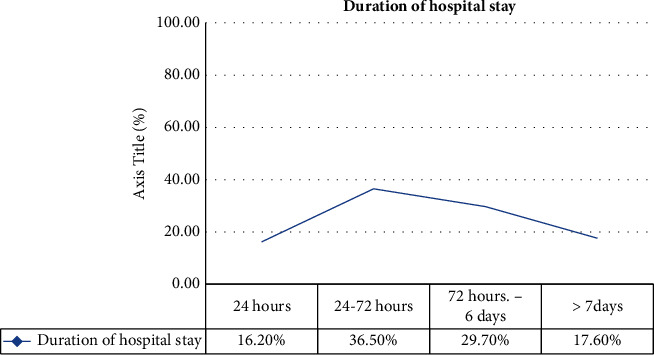
Duration of hospital stay of patients with hypertensive crisis presented to the EOPD at AHMC, Adama, Oromia, Ethiopia, from January 01 to August 31, 2021, G.C.

After presenting to the emergency room, 12 (16.2%) patients were discharged within 24 hours, 27 (36.5%) patients stayed for 24–72 hours (about 3 days), and 22 (29.7%) stayed for 6–72 days (Figure 8).

## 5. Discussion

### 5.1. Prevalence and Factors Associated with Hypertensive Crises

During the six-month duration of the study, 444 cases of hypertensive crisis were detected among the 9,082 patients attending the EOPD, indicating a prevalence of 4.9%. This figure closely resembles findings from a study conducted at Mulago Hospital's Emergency Department in Kampala, Uganda, which reported a prevalence of 5.1% [15].

In this study, men accounted for more than half (56.8%) of those diagnosed with hypertensive crisis, which is comparable to a study conducted in Brazil in 2000, which found that 55.3% were men and 44.7% were women [22].

Moreover, in this analysis, an increase in age above 55 years was found to be a significant risk factor for hypertensive crisis (*P*=0.004), as shown in the univariate analysis. These findings were similar to those studies conducted in the USA, where old age was one of the independent predictors of poorly controlled hypertension [23]. Similar results were obtained from a study conducted in Nigeria, where it was discovered that among black hypertensive patients who sought primary medical care, old age was most frequently linked to target organ damage [24].

Most of the patients (91.0%) with hypertensive crises were known hypertensive patients, and 40 (9.0%) of them were newly diagnosed. Only 39 (52.7%) were on follow-up, and 36 (48.6%) were found to be adherent; the major reason was a lack of knowledge, accounting for 41.4% of cases, and a sense of improvement in 28.1% of cases, particularly in rural areas where being far from the health center and cost were also factors. These studies are comparable with a prospective study performed on patients in the cardiac emergency ward of Imam Reza Hospital, which showed that 75% of them had discontinued taking their medication for a long time, with the most common reason being a feeling of improvement [25].

Salt consumption accounts for 234 (52.7%) of the risk factors for patients with hypertensive crises who presented to the emergency department in our study, followed by alcohol consumption (36.5%). Moreover, when we see the BMI of our study population, 107 (or 24.1%) of them are between 25 and 29.9 kg/m^2^. These are comparable with a population-based, cross-sectional survey performed in Addis Ababa, which showed that about 20% of males and 38% of females were overweight (body mass index ≥25 kg/m^2^). [26].

Furthermore, in the univariate analysis, our study found that a history of diabetes mellitus was a significant predictor of hypertensive crisis (*P*=0.002). This is similar to a study conducted in Brazil in which diabetes mellitus was found in 20% of the patients and was found to be a statistically significant factor for the development of hypertensive crisis [27]. Moreover, a study in Ghana showed that participants diagnosed with diabetes had increased odds of developing target organ damage compared to those with no diagnosis of diabetes [28].

Our study also found that patients from urban areas are significantly more associated with hypertensive emergencies and complications than those from rural ones. This can be explained by the current adoption of unhealthy cultures, including diet changes, smoking, obesity, and life changes associated with urbanization, all of which are associated with cardiovascular complications. While certain factors may indeed elevate the risk of developing hypertension in urban settings, recent research indicates that the prevalence of hypertensive emergencies may not exhibit a significant disparity between urban and rural areas. This observation may stem from the reality that rural regions often encounter challenges associated with limited access to healthcare services. Consequently, such limitations could potentially lead to an underestimation of the prevalence of hypertensive emergencies in rural areas [29]. Moreover, a study conducted in the Congo by Ellenga et al. revealed that hypertensive emergencies were significantly associated with low socioeconomic status [30].

### 5.2. Complications of a Hypertensive Crisis

In our study, stroke was a major complication, accounting for 28.6%. The number of emergencies from hemorrhagic stroke was 27.1%, which was higher than that in previous studies. Stroke was the most prevalent disease, accounting for 50% of the cases [31] in the study from the Democratic Republic of the Congo, whereas in the Italian study, stroke was responsible for 22% of the cases [7].

Furthermore, 8.2% of the patients had signs of heart failure contributing to cardiomegaly and left ventricular hypertrophy, and 4.1% had basal crepitation on chest examination. However, in the Italian study, 121 patients (30.9%) had acute pulmonary edema.

In Brazil, too, acute pulmonary edema was one of the most common hypertensive emergencies [27]. Our study has shown that 8.1% of patients with severe hypertension had renal dysfunction, which is lower than that in Congo, where renal failure was found in 13.1% of patients with severe hypertension [31], and again, this is lower than that in Italy, where 9.9% had acute renal failure [7]. These showed that hypertensive kidney diseases are undiagnosed because of the limited facilities in our setting.

### 5.3. Limitations of the Study

This study used a prospective cross-sectional survey obtained directly from the patient and secondary data from a single hospital. The result may not be representative of the national or regional picture as it is performed in one hospital involving a referral hospital population with a relatively well-organized surgical ward in the region. The diagnosis and patient condition were recorded on the clinical records. Due to costs, some confirmatory investigations, such as a CT scan and cardiac biomarkers, were not available in all cases.

In some cases, it was difficult to determine whether the end-organ damage in this study was caused by the currently severely elevated blood pressure, an old previous lesion, or some other chronic disease.

## 6. Conclusions

Hypertensive crises are one of the most common causes of admission among patients who visit the Emergency Outpatient Department (EOPD) and are associated with high rates of complications. Most of the patients were males between the ages of 66 and 75. More than half of the patients were from urban areas.

Factors commonly associated with hypertensive crises include age above 55 and the patient's place of residence. Most of the study subjects at the presentation were known hypertensive patients; they had been known to be hypertensive for 5–10 years. Less than half of the patients were found to be adherents, the major reason being a lack of knowledge.

Patients with hypertensive crises who presented to the emergency department had associated comorbid illnesses, and diabetes mellitus was found in a quarter of them.

The most common forms of hypertensive crisis include stroke, cardiac dysfunction, and renal dysfunction.

### 6.1. Public Health Implications

It is necessary for the government to place more emphasis on population-wide hypertension prevention and management. The potential complications of hypertension should be explained to patients. To provide hospitals with the necessary baseline investigations and to enhance the outcomes of hypertensive patients, infrastructure and capacity building are required.

Healthcare providers should educate patients on the importance of consistent use of medication on discharge to reduce the case fatality rate postdischarge. There is a need to educate the community on the importance of lifestyle modifications such as dietary modification and exercise to reduce the risk of hypertension and to develop a habit of checking their blood pressure for early detection and treatment to avoid the complications of hypertension, which are fatal. Patients should be evaluated and managed for possible records, including initial and follow-up laboratory investigation results.

## Figures and Tables

**Table 1:** Age, sex, and address of patients with hypertensive crisis presented to the EOPD at AHMC, Adama, Oromia, Ethiopia, from January 01 to August 31, 2021, G.C.

Variables		Frequency	Percentage
Age	<45 year	15	3.8
	45–55 year	67	15.1
	56–65 year	104	23.4
	66–75 year	190	42.8
	>75 year	78	17.6
	Total	444	100.0

Sex	Male	252	56.8
	Female	192	43.2
	Total	444	100.0

Place of Residency	Rural	97	21.8
	Urban	347	78.2
	Total	444	100.0

**Table 2:** History of patients with hypertensive crisis presented to the EOPD at AHMC, Adama, Oromia, Ethiopia, from January 01 to August 31, 2021, G.C.

Variables		Frequency	Percentage
Previously known hypertensive patients	Yes	404	91.0
	No	40	9.0
	Total	444	100.0

Duration of being hypertensive	<1 year	45	10.1
	1–5 year	141	31.8
	5–10 year	155	34.9
	>10 year	67	15.1
	Newly diagnosed	36	8.1
	Total	444	100.0

Were you on follow-up?	Yes	234	52.7
	No	174	39.2
	Newly diagnosed	36	8.1
	Total	444	100.0

Were you adherent to the medications?	Yes	216	48.6
	No	192	43.2
	Newly diagnosed	36	8.1
	Total	444	100.0

**Table 3:** Reasons for not being on follow-up of patients with hypertensive crisis presented to the EOPD at AHMC, Adama, Oromia, Ethiopia, from January 01 to August 31, 2021, G.C.

Variables		Frequency	Percentage
If you are not on follow-up, why?	Lack of knowledge	70	40.2
	Far from health center	34	19.5
	Negligence	34	19.5
	Feeling of being well	36	20.7
	Total	174	100.0

If you are not adherent, why?	Lack of knowledge	34	17.7
	Cost	54	28.1
	Negligence	25	13.1
	Fear of side effects	25	13.1
	Feeling of being well	54	28.1
	Total	192	100.0

**Table 4:** Current complaints of patients with hypertensive crisis presented to the EOPD at AHMC, Adama, Oromia, Ethiopia, from January 01 to August 31, 2021, G.C.

Variables		Frequency	Percentage
Current presenting complaint	Headache	195	43.9
	Body weakness	123	27.7
	Loss of consciousness	36	8.1
	Abnormal body movement	24	5.4
	Impaired vision	13	2.9
	Incidental on follow-up	53	12.0
	Total	444	100.0

Duration of complaint	<24 hour	162	36.5
	24–72 hour	121	27.2
	3–7 days	65	14.7
	1–2 weeks	30	6.8
	>2 weeks	10	2.3
	Incidental finding	56	12.6
	Total	444	100.0

**Table 5:** Physical findings of patients with hypertensive crisis presented to the EOPD at AHMC, Adama, Oromia, Ethiopia, from January 01 to August 31, 2021, G.C.

Variables		Frequency	Percentage
Blood pressure at presentation	180/110–190/115	222	50.0
	191/116–200/120	137	30.9
	201/121–220/130	61	13.7
	>220/130	24	5.4
	Total	444	100.0

BMI	<18.5 kg/m^2^	30	6.8
	18.5–24.9 kg/m^2^	199	44.8
	25–29.9 kg/m^2^	107	24.1
	30–34.9 kg/m^2^	66	14.9
	35–39.9 kg/m^2^	30	6.8
	>40 kg/m^2^	12	2.7
	Total	444	100.0

Cardiac finding	Displaced apex	30	6.8
	No finding	414	93.2
	Total	444	100.0

Neurologic finding (GCS)	15/15	270	60.8
	13–14	54	12.2
	8–12	84	18.9
	<8	36	8.1
	Total	444	100.0

**Table 6:** Laboratory findings of patients with hypertensive crisis presented to the EOPD at AHMC, Adama, Oromia, Ethiopia, from January 01 to August 31, 2021, G.C.

Variables		Frequency	Percentage
FBS/RBS	<70 mg/dl	30	6.8
	70–110 mg/dl (normal)	331	74.5
	111–200 mg/dl	59	13.3
	>200 mg/dl	24	5.4
	Total	444	100.0

Serum creatinine	0.7–1.2 mg/dl (normal)	366	82.4
	1.3–2.0 mg/dl	53	11.9
	>2.0 mg/dl	25	5.6
	Total	444	100.0

Alanine Transaminase	0–40 U/L (normal)	341	76.8
	41–100 U/L	36	8.1
	100–200 U/L	24	5.4
	>200 U/L	43	9.7
	Total	444	100.0

Aspartate Transaminase	0–40 U/L (normal)	347	78.1
	41–100 U/L	30	6.8
	100–200 U/L	37	8.3
	>200 U/L	30	6.8
	Total	444	100.0

Triglycerides	0–150 mg/dl (normal)	360	81.1
	150–200 mg/dl	40	9.0
	>300 mg/dl	44	10.0
	Total	444	100.0

Cholesterol	0–200 mg/dl (normal)	354	79.7
	200–300 mg/dl	49	11.0
	>250 mg/dl	41	9.2
	Total	444	100.0

**Table 7:** Radiologic findings of patients with hypertensive crisis presented to the EOPD at AHMC, Adama, Oromia, Ethiopia, from January 01 to August 31, 2021, G.C.

Variables		Frequency	Percentage
CXR findings	Normal	250	56.3
	Pulmonary edema	19	4.3
	Cardiomegaly	11	2.5
	Infiltration (SCAP)	30	6.8
	Not performed	134	30.1
	Total	444	100.0

ECG findings	Normal	378	85.1
	STEMI	16	3.6
	Nonspecific ST depression	4	0.9
	Left ventricular hypertrophy	18	4.1
	Not performed	30	6.8
	Total	444	100.0

Echocardiography findings	Normal	377	84.9
	Dilated cardiomyopathy	13	2.9
	Ischemic cardiomyopathy	6	1.4
	Ischemic heart disease	19	4.3
	Not performed	29	6.6
	Total	444	100.0

CT scan findings	Normal CT scan	77	17.4
	Haemorrhagic stroke	73	16.4
	Ischemic stroke	54	12.2
	Not performed	240	54.1
	Total	444	100.0

## Data Availability

The data used to support the findings of this study are available from the corresponding author upon request, provided that data sharing agreements and ethical considerations are met.

## Abbreviations

ACC: American College of Cardiology
AHA: American Heart Association
AHMC: Adama Hospital Medical College
MAP: Mean arterial pressure
BMI: Body mass index
BP: Blood pressure
CHD: Coronary heart disease
CHF: Congestive heart failure
CKD: Chronic kidney disease
CT: Computed tomography
CXR: Chest X-ray
DBP: Diastolic blood pressure
ECG: Electrocardiography
EOPD: Emergency Outpatient Department
FBS: Fasting blood sugar
HIV: Human immunodeficiency virus
HTN: Hypertension
ISH: International Society of Hypertension
JNC: Joint National Committee
NCHS: National Center for Health Statistics
RBS: Random blood sugar
SSA: Sub-Saharan Africa
SBP: Systolic blood pressure
WHO: World Health Organization.

## Supplementary Materials

Supplementary Materials S_1: *data collection format*. Questionnaire: a prospective cross-sectional survey to study the prevalence and risk factors of hypertensive crisis in patients who visited the Emergency Outpatient Department (EOPD) at Adama Hospital Medical College, Adama, Oromia, Ethiopia, from August 10 to August 30, 2021, G.C.

## References

[1] Kabore J., Mounkaila F., Yanogo P., Laurent M., Otshudiandjeka J., Meda N. (2022). Prevalence and factors associated with cardiovascular emergencies in the emergency department of niamey national hospital. *Health Sciences and Disease*.

[2] James P. A., Oparil S., Carter B. L. (2014). 2014 evidence-based guideline for the management of high blood pressure in adults: report from the panel members appointed to the Eighth Joint National Committee (JNC 8). *JAMA*.

[3] Baurenski L., Trendafilova E. (2018). Blood Pressure reduction and outcome in patients with hypertensive crisis and acute ischemic stroke. *Journal of Hypertension*.

[4] Katz J. N., Gore J. M., Amin A. (2009). Practice patterns, outcomes, and end-organ dysfunction for patients with acute severe hypertension: the Studying the Treatment of Acute hyperTension (STAT) Registry. *American Heart Journal*.

[5] World Health Organization *A Global Brief on Hypertension: Silent Killer, Global Public Health Crisis: World Health Day 2013*.

[6] Kearney P. M., Whelton M., Reynolds K., Muntner P., Whelton P. K., He J. (2005). Global burden of hypertension: analysis of worldwide data. *The Lancet*.

[7] Pereira M., Lunet N., Azevedo A., Barros H. (2009). Differences in prevalence, awareness, treatment and control of hypertension between developing and developed countries. *Journal of Hypertension*.

[8] Kotchen T. A. (2023). Hypertensive vascular disease. *Harrison’s Principles of Internal Medicine*.

[9] Almas A., Ghouse A., Iftikhar A. R., Khursheed M. (2014). Hypertensive crisis, burden, management, and outcome at a tertiary care center in karachi. *International Journal of Chronic Diseases*.

[10] Aggarwal M., Khan I. A. (2006). Hypertensive crisis: hypertensive emergencies and urgencies. *Cardiology Clinics*.

[11] Varon J., Marik P. E. (2003). Clinical review: the management of hypertensive crises. *Critical Care*.

[12] Desta D. M., Wondafrash D. Z., Tsadik A. G. (2020). Prevalence of hypertensive emergency and associated factors among hospitalized patients with hypertensive crisis: a retrospective cross-sectional study. *Integrated Blood Pressure Control*.

[13] Shao P. J., Sawe H. R., Murray B. L., Mfinanga J. A., Mwafongo V., Runyon M. S. (2018). Profile of patients with hypertensive urgency and emergency presenting to an urban emergency department of a tertiary referral hospital in Tanzania. *BMC Cardiovascular Disorders*.

[14] Onggo S. I., Wiharja W., Bertha S. N., Sutisna N., Taslim A. (2018). 2 Clinical profile and epidemiology of hypertensive crisis at emergency unit in Cilegon, Banten. *Journal of Hypertension*.

[15] Nakalema I., Kaddumukasa M., Nakibuuka J., Okello E., Sajatovic M., Katabira E. (2019). Prevalence, patterns and factors associated with hypertensive crises in Mulago hospital emergency department: a cross-sectional study. *African Health Sciences*.

[16] Ferguson J. L., Beckett G. J., Stoddart M., Walker S. W., Fox K. A. (2002). Myocardial infarction redefined: the new ACC/ESC definition, based on cardiac troponin, increases the apparent incidence of infarction. *Heart*.

[17] Park J. H., Kim S. M., Shin H. W., An S. J. (2010). Hypertensive brainstem encephalopathy involving deep supratentorial regions: does only blood pressure matter?. *Neurology International*.

[18] Murray J. F. (2011). Pulmonary edema: pathophysiology and diagnosis. *International Journal of Tuberculosis and Lung Disease: The Official Journal of the International Union Against Tuberculosis and Lung Disease*.

[19] Barile M. (2020). Pulmonary edema: a pictorial review of imaging manifestations and current understanding of mechanisms of disease. *European journal of radiology open*.

[20] Henderson A. D., Bruce B. B., Newman N. J., Biousse V. (2011). Hypertension-related eye abnormalities and the risk of stroke. *Reviews in Neurological Diseases*.

[21] Ojaghihaghighi S., Vahdati S. S., Mikaeilpour A., Ramouz A. (2017). Comparison of neurological clinical manifestation in patients with hemorrhagic and ischemic stroke. *World journal of emergency medicine*.

[22] Pollack C. V. J., Rees C. J. (2009). Hypertensive emergencies: acute care evaluation and management. *EMCREG-International*.

[23] Janke A. T., McNaughton C. D., Brody A. M., Welch R. D., Levy P. D. (2016). Trends in the incidence of hypertensive emergencies in US emergency departments from 2006 to 2013. *Journal of the American Heart Association*.

[24] Oladapo O. O., Salako L., Sadiq L., Shoyinka K., Adedapo K., Falase A. O. (2012). Target-organ damage and cardiovascular complications in hypertensive Nigerian Yoruba adults: a cross-sectional study: cardiovascular topics. *Cardiovascular Journal of Africa*.

[25] Rafighdoust A., Mohammadzadeh Shabestari M., Bostani T. (2006). A study of hypertensive crisis and precipitating factors. *Iranian Heart Journal*.

[26] Tesfaye F., Byass P., Wall S. (2009). Population based prevalence of high blood pressure among adults in Addis Ababa: uncovering a silent epidemic. *BMC Cardiovascular Disorders*.

[27] Papadopoulos D. P., Sanidas E. A., Viniou N. A. (2015). Cardiovascular hypertensive emergencies. *Current Hypertension Reports*.

[28] Knight E. L., Bohn R. L., Wang P. S., Glynn R. J., Mogun H., Avorn J. (2001). Predictors of uncontrolled hypertension in ambulatory patients. *Hypertension*.

[29] Ranzani O. T., Kalra A., Di Girolamo C. (2022). Urban-rural differences in hypertension prevalence in low-income and middle-income countries, 1990-2020: a systematic review and meta-analysis. *PLoS Medicine*.

[30] Ellenga M. B., Gombet T. R., Mahoungou G. K. (2011). Les urgences hypertensives au Centre Hospitalier et Universitaire de Brazzaville (Congo). *Medecine Tropicale: Revue du Corps de Sante Colonial*.

[31] Mensah G. A. (2002). The global burden of hypertension: good news and bad news. *Cardiology Clinics*.

[32] Abebe A. T., Kebede Y. T., Mohammed B. D. (2023). An assessment of the prevalence and risk factors of hypertensive crisis in patients who visit the Emergency Outpatient Department (EOPD) at Adama Hospital Medical College, Adama, Oromia, Ethiopia: a 6-month prospective study. https://www.x-mol.net/paper/article/1698049859900952576.

