# Supplementary material for: An Assessment of the Prevalence and Risk Factors of Hypertensive Crisis in Patients Who Visited the Emergency Outpatient Department (EOPD) at Adama Hospital Medical College, Adama, Oromia, Ethiopia: A 6-Month Prospective Study
Source: Int J Hypertens. 2024 Apr 29;2024:6893267. doi: 10.1155/2024/6893267 (PMC11073854; doi:10.1155/2024/6893267)
Supplement: Supplementary Materials — S_1: data collection format. Questionnaire: a prospective cross-sectional survey to study the prevalence and risk factors of hypertensive crisis in patients who visited the Emergency Outpatient Department (EOPD) at Adama Hospital Medical College, Adama, Oromia, Ethiopia, from August 10 to August 30, 2021, G.C. [file 6893267.f1.docx]

# **Data Collection Format**

Questionnaire: A prospective cross-sectional survey to study the prevalence and risk factors of Hypertensive crisis in patients who visit the Emergency Outpatient Department (EOPD) at Adama Hospital Medical College, Adama, Oromia, Ethiopia, from August 10 to August 30, 2021, G.C’

Instructions to the interviewer:

Mark on the space provided (√)

Explain the purpose of the study to the interviewers and obtain verbal consent.

**I. Socio-demographic characteristics**

1. Age_____

2. Sex

A. Male □ B. Female □

3. Place of residence

A. Rural…□ … B**.** Urban □ ……

4. Educational level

A. Illiterate □ D. read & write □

B. Primary (grades 1-8) □ E. Secondary (9-12) □

C. Secondary and higher

5. Occupation

A. Government employee □ D. Farmer □

B. Private employee □ E. Housewife □

C. Merchant □ F. Other _____

6. Income per month

A. < 3880 birr □ C. 15136 – 46,900 birr □

B. 3880 – 15,135 birr □ D. >46,900 birr □

**NB:** This is according to the World Bank classification of 2020–2021 GNI per capita calculated using the World Bank Atlas method per family income summed up. (33)

**II. Previous history**

1. Previously known hypertensive?

A. Yes □ B. No □

2. If yes to question no. 1, the duration of being hypertensive

A. < 1 year □ C. 5-10 years □

B. 1-5 years □ D. > 10 years □

3. Were you on the follow-up list if you answered yes to question 2?

A. Yes □ B. No □

4. If no to question 3, why?

A. ignorance; C. negligence; B. living far from a health facility; D. other, specifying _____.

5. If yes to question 3, in which health institution?

B. Hospital □ C. Private Clinic □

C. Health center □ D. Other, specify _______

6. If yes to question no. 4, were you adherent to the medications?

A. Yes □ B. No □

7. If no to question 6, why?

A. Lack of knowledge □ D. Fear of side effects □

B. Cost □ E. other, specify_______

C. Negligence □

8. If yes to question 6, medications being taken on follow up

B. Diuretics E. ACE Inhibitors

- Lasix □ - Captoril □

- Hydrochlorothiazide □ - Enalapril □

C. Calcium channel blockers

- Amlodipine □ - Nifedipine □

D. Beta blockers

- Atenolol - Propranolol □

E. Other, specify_______

9. If yes to question 8, the duration of treatment

A. < 1 year □ C. 5-10 years □

B. 1-5 years □ D. > 10 years □

III. **The current complaint**

1. Current presenting symptom

A. Headache □ F. Shortness of breath □

B. Loss of consciousness □ G. Chest pain □

C. Abnormal body movement □ H. Incidental on follow-up □

D. Impaired vision □ I. Others specify_______________

E. Neck pain □

2. Duration of compliance

A. 24 hour □ D. 1 to 2 weeks □

B. 24 to 72 hours □ E. > 2 weeks □

C. 3 to 7 days □

3. Associated compliant

A. Headache □ F. Shortness of breath □

B. Loss of consciousness □ G Chest pain □

C. Abnormal body movement □ H. Decreased urine output

D. Impaired vision □ I. Others specify_______________

E. Neck pain □

IV. **Any identified risk factors for hypertensive crises?**

1. Do you have a family history of hypertension?

A. Yes □ B. No □

2. If yes to question 1, which family member?

A. 1^st^ degree relative; specify______

B. 2nd-degree relative

3. Do you use alcohol for recreational purposes?

A. Yes □ B. No □

4. If yes to Q 3, how many bottles of alcohol per day? __________

5. If yes to Q 3, how many days a week? _________

6. Do you smoke a cigarette?

A. Yes □ B. No □

7. If yes to Q 6, how many cigarettes a day? _________

8. Do you chew “chat”?

A. Yes □ No □

9. If yes to Q 8, how many days in a week? _________

10. Do you do regular physical exercise?

A. Yes □ No □

11. If yes to Q 10, how often do you do regular physical exercise?

A. Daily □ D. 2-3 times/ week □

B. 4–5 times/ week □ E. weekly □

12. Do you consume salt?

A. Yes □ B. No □

13. How often do you consume:

A. Vegetables_______

B. Fatty food _______

C. Other specify_______

14. Previous history of severe hypertensive range?

Yes □ No □

15. Other Co-morbid Illnesses

A. Diabetes Mellitus□ C. Renal failure □

B. HIV/AIDS □ D. Metabolic syndrome □

C. CHF □ E. Others, specify__________

D. No Co-morbidity □

V. **Physical examination**

1. Blood pressure at presentation______

2. Weight in Kg______, Height______, BMI______

3. Respiratory finding

A. Crepitations □ C. Other__________

B. Decrease air entry □ D. No finding □

4. Cardiac finding

A. Elevated JVP □ C Other__________

B. Displaced apex □ D. No finding □

5. Abdominal finding

A. Organomegally □ C. Other___________

B. Sign of fluid collection □ D. No finding □

6. Musculo-skeletal finding

A. Bilateral leg edema C. No finding □

B. other__________

7. Neurologic finding

A. GCS_______

B. Motor examination: Muscle tone_______

: Muscle power_______

: Reflex _______

C. Other_______

D. No finding□

VI. **Investigations done**

1. FBS/RBS_______

2. Complete blood count

A. White blood count_______

B. Hemoglobin_______

3. Urinalysis

A. Glucose_______ C. Albumin_______

B. Ketone_______ D. Microscopy_______

4. Renal function test

A. Serum Creatinine _______

B. Urea_______

5. Liver function test

A. Alanine Transaminase_______

B. Aspartate Transaminase _______

C. Alkaline phosphatse_______

6. Lipid profile

A. Triglycerides_______ C. LDL_______

B. Cholesterol_______ D. HDL_______

7. CXR findings

A. Normal □ C. Cardiomegally □

B. Pulmonary edema □ D. Other_______

8. ECG findings

A. Normal □ D. Left ventricular hypertrophy □

B. STEMI □ E. Other_______

C. Non-specific ST depression □

9. Echocardiography findings

A. Normal □ C. Thrombus □

B. Murmur □ D. Other_______

10. CT scan findings( if done)

A. Normal CT scan □ C. Ischemic stroke □

B. Hemorrhagic stroke □ D. Other _______

VII. **Diagnosis**

1. Hypertensive urgency □ 2. Hypertensive emergency □

VIII. **Medication**

1. Medication given

A. Diuretics F. ACE Inhibitors

- Lasix □ - Captoril □

- Hydrochlorothiazide □ - Enalapril □

B. Calcium channel blockers

- Amlodipine □ - Nifedipine □

C. Beta blockers

- Atenolol - Propranolol □

D. Hydralazine □

E. Other, specify_______

2. Route of administration

A. Intravenous □. B. Oral □ C. Sublingual □

3. Frequency

A. Once a day □ C. Three times a day □

B. Twice a day □ D. ≥ Four times a day □

4. Target BP after

A. 6 hours for a hypertensive emergency_________

B. 36 hours for a hypertensive urgency__________

IX. **Duration of the hospital stay**

1. 24 hours □ 3. 72 hours. – 6 days □

2. 24-72 hours □ 4. > 7days □

X. **Outcome**

1. Improved □ 4. Left against medical advice □

2. Deteriorate □ 5. Unknown □

3. Expired □

Name of interviewer ____________________________

Signature __________ Date __________
